# Frozen and Scorched: Genomic Signatures of Adaptive Divergence and Hibernation in the Living Fossil *Dromiciops*

**DOI:** 10.1002/ece3.74066

**Published:** 2026-08-02

**Authors:** Elisa Gonzalez‐Ugalde, Paula M. Avendaño, Julián F. Quintero‐Galvis, Eduardo J. Pizarro, Francisco A. Cubillos, Roberto F. Nespolo, Fabiola León, Juliana A. Vianna

**Affiliations:** ^1^ Pontificia Universidad Católica de Chile Facultad de Ciencias Biológicas Santiago Chile; ^2^ Millennium Institute Center for Genome Regulation (CGR) Santiago Chile; ^3^ Millennium Nucleus of Patagonian Limit of Life (LiLi) Valdivia Chile; ^4^ Millennium Institute Biodiversity of Antarctic and Subantarctic Ecosystems (BASE) Santiago Chile; ^5^ Departamento de Ciencias, Facultad de Artes Liberales Universidad Adolfo Ibáñez Santiago Chile; ^6^ Facultad de Química y Biología, Departamento de Biología Universidad de Santiago de Chile Santiago Chile; ^7^ Millennium Institute for Integrative Biology Santiago Chile; ^8^ Instituto de Ciencias Ambientales y Evolutivas Universidad Austral de Chile Valdivia Chile; ^9^ Center of Applied Ecology and Sustainability (CAPES), Facultad de Ciencias Biológicas Universidad Católica de Chile Santiago Chile; ^10^ One Health Institute, Faculty of Life Sciences Universidad Andrés Bello Santiago Chile

**Keywords:** adaptation, comparative genomics, marsupial, selection, torpor

## Abstract

Adverse historical conditions shape biodiversity by driving demographic shifts and genomic divergence through evolutionary mechanisms. Selective pressures foster adaptive strategies in response to extreme environments, with hibernation as a key example. The expression of torpor and its various forms exemplify such adaptations. However, the genomic basis of these processes and the influence of evolutionary forces, particularly natural selection, remain largely unexplored. The Monito del Monte (*Dromiciops*), a small marsupial considered a living fossil, exhibits torpor, hibernation, and aestivation as key survival strategies. In this study, we investigated genomic adaptations in *Dromiciops* and their potential relationship with the physiological state of torpor and hibernation. Historical climate change, along with varying expansions and bottlenecks, appears to have shaped the genetic diversity and local adaptation of *Dromiciops*. At the ancestral *Dromiciops* node, positive selection was detected in *PFKFB2, LAMP3, OTX1*, and *RAPSN*, alongside enrichment in the MAPK signaling and steroid hormone biosynthesis pathways—both previously implicated in hibernation physiology. Gene family expansions further converged on mitochondrial maintenance and redox regulation. At the species level, *D. gliroides* and *D. bozinovici* show no overlap in positively selected genes: *D. gliroides* presents additional signals in LSS and RASL11B, related to sterol and stress‐response metabolism, while *D. bozinovici* shows signatures of broad thermal stress tolerance through HSPA2. These findings underscore unique adaptations possibly linked to hibernation in *Dromiciops* and species‐specific differences.

## Graphical abstract

We evaluated adaptation to torpor and hibernation in the genus *Dromiciops* by comparing it with both torpid and non‐torpid marsupials, and investigated divergent selection signals between its two species. For the ancestral node, we identified positive selection in the genes PFKFB2, LAMP3, OTX1, and RAPSN, alongside an enrichment of pathways potentially linked to hibernation, which aligns with our gene family expansion findings. No overlap of genes under selection was found between species; specific signals were identified in LSS and RASL11B for *D. gliroides*, and in HSPA2 for *D. bozinovici*.

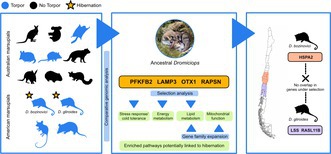

## 1 Introduction

The evaluation of positive selection is particularly compelling in regions with dynamic environmental histories, such as the South American temperate forests. The organisms inhabiting these forests have been profoundly shaped by glacial and interglacial periods, which brought about drastic environmental changes (Núñez‐Ávila and Armesto 2006). During glacial periods, extensive ice coverage dramatically transformed the landscape and restricted the development of floristic communities, particularly forest ecosystems (Villagran 1990; Heusser et al. 2006), thereby imposing new selective pressures on the organisms inhabiting these environments. Fragmentation, isolation, and exposure to novel environmental conditions likely triggered diversification processes across multiple taxa, ultimately contributing to the region's high levels of endemism (Smith‐Ramírez 2004). Among these environmental factors, temperature was one of the most influential, undergoing severe fluctuations throughout the glaciations (Heusser 2003).

The selective pressures exerted by contrasting climatic conditions during extreme climatic periods likely played a pivotal role in shaping species divergence (Lister 2004). In such environments, adaptations to withstand severe cold and increasingly seasonal or fragmented food availability likely became essential strategies for survival (Lister 2004). One possible adaptation that may have emerged as a result of extreme environmental conditions is torpor. Torpor is defined as a state in which endotherms exhibit controlled and significant reductions in metabolic rate and body temperature (Ruf and Geiser 2015), which is triggered by environmental cues such as temperature fluctuations, resource scarcity, and unpredictable challenging conditions (Nowack et al. 2015). Theories on the evolution of torpor propose it as either a convergent trait or an ancestral characteristic (Geiser 2021; Weir et al. 2024), which may have facilitated survival and promoted the selection of more complex endothermic capabilities in extreme environments (Geiser 2020, Geiser 2021). Based on torpor duration, mammals and birds are classified as either daily heterotherms or hibernators (Ruf and Geiser 2015). Hibernators experience multiday bouts lasting over a week, while daily heterotherms undergo shorter bouts lasting less than a day (Geiser and Ruf 1995). Additionally, some species undergo aestivation, a hypometabolic state typically triggered by high temperatures and water shortage (Jiang et al. 2023). Although the genomic processes underlying hibernation have been more extensively studied than those associated with daily torpor (Shankar et al. 2023), both states share common mechanisms, which include a metabolic shift from carbohydrates to lipids, maintenance of cellular homeostasis, proteolysis, and protection against oxidative stress (Andrews 2019). The genomic basis of torpor and hibernation have primarily been investigated mainly through transcriptomic analyses (Fedorov et al. 2014; Jansen et al. 2019; Nespolo et al. 2018), with few works evaluating the possible role that positive selection may exert in the phenotype (Villanueva‐Canas et al. 2014).

Torpor is a widespread adaptive strategy among mammals, occurring across various lineages (Kontopoulos et al. 2025). In the case of marsupials, torpor is present in both American and Australian species. As reviewed by Geiser and Cooper (2023), the expression of heterothermy is highly variable across lineages; in America, species capable of entering torpor are found across all three recognized orders (Didelphimorphia, Microbiotheria, and Paucituberculata). Conversely, within the four strictly Australian orders, heterothermic species are distributed across three of them (Dasyuromorphia, Notoryctemorphia, and Diprotodontia). Although daily torpor is widely documented across both geographical regions, deep prolonged hibernation is predominantly restricted to specific Australian families (Geiser and Cooper 2023). The South American genus *Dromiciops*, which has been identified as the sister lineage to all Australian marsupials (Feng et al. 2022), stands out as a notable exception, uniquely exhibiting not only hibernation and daily torpor, but also aestivation (Nespolo et al. 2022). The genetic basis of hibernation in this genus has been primarily explored through gene expression analyses, identifying pathways such as mTOR, MAPK, and Akt signal transduction as central to metabolic regulation and homeostasis maintenance during hibernation (Nespolo et al. 2018; Luu et al. 2018; Wijenayake et al. 2018). Similar regulatory pathways have been identified in other hibernating mammalian species (Zhu et al. 2005; Eddy and Storey 2007), further supporting the significance of these mechanisms in adaptive responses to environmental stress.

Currently, there are two recognized species of *Dromiciops* known as the monito del monte; *D. bozinovici* and *D. gliroides* ; the latter is subdivided into two subspecies *D. g. mondaca* (north) and *D. g. gliroides* (south) (Quintero‐Galvis et al. 2021, 2022; D'Elia et al. 2016). *Dromiciops* is distributed across Chile and Argentina, spanning a wide latitudinal range. In Chile, its distribution extends from the Maule region (35°50′ S, 72°30′ W) to the locality of Chaitén in the Los Lagos Region (45°00′ S, 72°00′ W) (Oda et al. 2019). Although both species are found within the Valdivian temperate rain forests, *D. bozinovici* occupies the northern part of this range, primarily found in the regions of Maule, BíoBío, and La Araucanía (35.2° S to 39.3° S) (D'Elia et al. 2016; Quintero‐Galvis et al. 2021), while *D. gliroides* inhabits the southern regions. *D. bozinovici* can be found not only in evergreen forests but also in deciduous and sclerophyllous forests (Lobos et al. 2005), the latter characterized by hot, dry summers and mild, wet winters (Lionello et al. 2006). The Valdivian temperate rainforests, where *D. gliroides* predominantly occurs, experience high levels of rainfall year‐round and are dominated by evergreen species. *D. gliroides* distribution was historically impacted by glacial cycles, such as the extent of ice masses during the Last Glacial Maximum (LGM) (Heusser 2003). A recent study by Quintero‐Galvis et al. (2024) using RADseq data identified candidate loci under selection within each species, with allelic frequencies associated with precipitation and temperature variables across their respective ranges.

This study aims to explore potential genomic signatures associated with torpor and hibernation in *Dromiciops*, and to characterize patterns of genomic differentiation between its two extant species, using high‐coverage genomes (~20×) for intra‐ and interspecific comparisons. By examining patterns of positive selection and gene family dynamics, we investigate whether these signals may reflect lineage‐specific adaptations to hibernation, and whether the two species show distinct molecular trajectories consistent with divergent selective pressures.

## 2 Methods

### 2.1 Sampling

**FIGURE 1:**
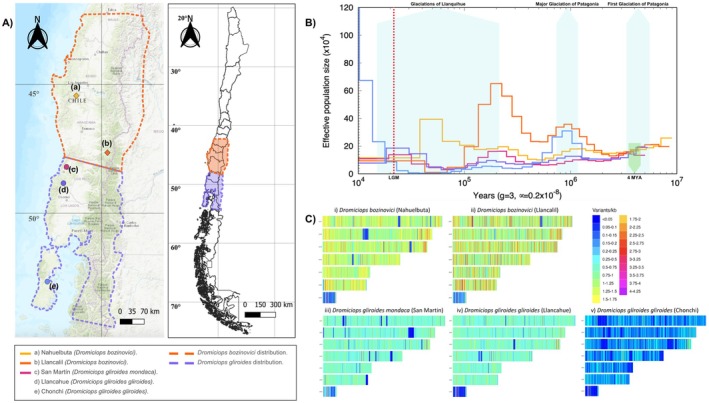
(A) Map of southern South America and Chile, depicting five localities where the specimens of *Dromiciops* included in this study were collected. Shape and color of the locality and distribution differ among the two species (*Dromiciops bozinovic*i; orange and *Dromiciops gliroides* purple) (Quintero‐Galvis et al. 2021). Localities were enumerated latitudinally from north to south (a to e are color coded for 1a and 1b). (B) Trajectories of effective population size (*N*_e_) for both species inferred using the PSMC model. The red dashed line indicates the Last Glacial Maximum (LGM). Shaded regions represent major glaciation periods, and the green arrow indicates the estimated diversification time. *N*_e_ trajectories were scaled using a generation time of 3.0 years and a mutation rate of 1.54 × 10^−9^ (Quintero‐Galvis et al. 2021). (C) SNP density across chromosome‐length scaffolds for individuals of (i) *D. bozinovici* (Nahuelbuta), (ii) *D. bozinovici* (Llancalil), (iii) *D. gliroides mondaca* (San Martín), (iv) *D. gliroides gliroides* (Llancahue), and (v) *D. gliroides gliroides* (Chonchi), based on the chromosome‐length assembly of *D. gliroides* .

Tissues (skeletal muscle, ear skin, and liver) were obtained from specimens stored at −80°C in a long‐established tissue bank from the Universidad Austral de Chile. This tissue bank was developed over several years in collaboration with public institutions, including the Agricultural and Livestock Service (SAG) and the National Forestry Corporation (CONAF), using samples from animals found dead in firewood piles or as a result of attacks by domestic dogs or cats. For this study, tissues from two specimens of *Dromiciops bozinovici* and three of *Dromiciops gliroides* were used, representing a total of five locations across southern Chile (Figure 1A; Table S1). *D. gliroides* belonged to the three clades proposed by Quintero‐Galvis et al. (2022). The tissue samples were preserved in 96% ethanol and transported to Santiago, Chile, for DNA extraction.

### 2.2 DNA Isolation and Genome Sequencing

DNA from each specimen was isolated using a salt extraction protocol (Aljanabi and Martinez 1997) with slight modifications (Vianna et al. 2017). Quantification was then performed using the QubitTM dsDNA BR Assay Kit and the Life Technologies Qubit fluorometry system (Thermo Fisher Scientific). The integrity of the extracted DNA was verified through agarose gel electrophoresis at 1%. Once these two quality parameters were verified, the extracted DNA was delivered to MedGenome (USA) for sequencing. The sequencing was performed using Illumina HiSeq technology, resulting in approximately 55 Gb of data per sample and ~20× depth coverage.

### 2.3 Quality Control and Alignment

The quality of the raw sequences was initially assessed using FastQC (Andrews 2010), followed by a summary report generated with MultiQC (Ewels et al. 2016). Adapter removal and quality trimming of the reads were conducted using Trimmomatic (Bolger et al. 2014), applying filters to remove low‐quality bases (min read length of 36, among others) and retain only high‐quality reads. The trimmed sequences were then aligned to the RefSeq genome of a female *D. gliroides* using the BWA‐MEM algorithm (Li 2013). The reference genome (mDroGli1.pri, NCBI RefSeq assembly GCF_019393635.1) (Rhie et al. 2021), which is assembled at the chromosomal level and has an annotation file (GFF), was retrieved from the GenBank database. After alignment, the SAM files were converted to BAM format and then sorted using Samtools (Li et al. 2009). Duplicate reads were identified and marked with the MarkDuplicates tool from Picard (Broad Institute), ensuring that duplicate artifacts from the sequencing process were excluded from downstream analyses. Additionally, Samtools was used to filter out reads with low mapping quality (MAPQ < 20).

### 2.4 Variant Calling

A SNP calling pipeline was implemented to enable downstream analyses of genomic diversity and adaptation. SNP calling was performed in two steps. First, bcftools (Li et al. 2009) was used to generate raw variant information from the deduplicated and sorted BAM files, which contained aligned sequences from all samples. Variants were then called with bcftools call, applying a minimum base quality filter of 20 and skipping indels. Post‐calling, the VCF files were compressed and indexed with bgzip and tabix, both commands from bcftools (Li et al. 2009). The resulting VCFs were further filtered using vcftools (Danecek et al. 2011) to retain only high‐quality variants, with filters set for minimum quality (*Q* ≥ 30), minimum read depth (DP ≥ 3), and allowing up to 10% missing data per site. A consensus FASTA sequence was then generated for each sample using bcftools consensus with default settings for subsequent selection analysis. Finally, regions with insufficient coverage (coverage < 1) were masked using bedtools (Quinlan and Hall 2010).

### 2.5 Demographic History

Demographic fluctuations in *Dromiciops* species were analyzed to assess whether past changes in population size could be linked to historical climatic events and to explore how such fluctuations may have influenced genomic diversity and the action of natural selection over time. To reconstruct the long‐term demographic history, ancestral inference was performed using the Pairwise Sequentially Markovian Coalescent (PSMC) model version 0.6.5‐r67 (Li and Durbin 2011). BAM files were processed through a pipeline, first using SAMtools version 1.3.1 (Li et al. 2009) to generate a consensus diploid FASTQ file. This file was then converted into PSMC input format using the fq2psmcfa command, and the resulting file was analyzed with the following parameters: ‐N25 ‐t15 ‐r5 ‐p “4+25*2+4+6.” Following PSMC documentation and Li and Durbin (2011), the minimum read depth (−d) was set to one‐third of the average coverage, and the maximum depth (−D) at most twice the average. To reduce biases caused by sex‐linked variation, sex chromosomes and coding sequences (CDS) were removed. CDS coordinates were obtained from genome annotations in GFF format and converted to BED format. These regions were filtered from the .psmcfa input files using SeqKit subseq (Shen et al. 2016). This step ensured a better fit with the assumptions of neutrality inherent to the PSMC model. Before performing bootstrap analyses, the input sequences were fragmented using the splitfa function to simulate genome‐wide sampling variation (Li and Durbin 2011). Multiple pseudo‐replicates were then generated to estimate confidence intervals using the same PSMC parameters. A mutation rate of 1.54 × 10^−9^ and a generational time of 3 years were used for the reconstructions (Quintero‐Galvis et al. 2022; Feng et al. 2022).

While PSMC is well suited for inferring ancient demographic trends over hundreds of thousands of years, it lacks resolution for more recent events. To complement this, the effective population size from recent generations was evaluated using GONE software (Novo et al. 2023). This evaluation involved linkage disequilibrium analysis at genomic marker loci to infer the effective population size trajectories over a period of about 100–200 generations back in time. To accomplish this, PLINK (Purcell et al. 2007) was used to generate the MAP and PED files. Subsequently, the effective population size was inferred for each generation by chromosome. Since GONE requires at least two individuals to infer recent demography, the two available genomes of *D. bozinovici* were used to assess effective population sizes in recent generations.

### 2.6 SNP Density Analysis

To investigate how genetic diversity is distributed across the genome and between the *Dromiciops* species, a SNP density analysis was performed by estimating the distribution of heterozygous single nucleotide polymorphisms (SNPs). This approach provides a proxy for local genomic diversity and allows the identification of regions with unusually high or low variation, which may reflect the action of selective pressures or historical demographic events. The SNP density plots were generated for each *Dromiciops* sample based on the final VCF file using VCFtools “snpden” (Danecek et al. 2011) function with a window size of 1 Mb, and a custom script in R (https://github.com/henriquevf/snpden_plot). These SNP densities were then plotted onto chromosome‐level assemblies for each species and location to assess the genomic composition of the variants, with the results scaled to SNPs per Kbp (Figure 1C).

### 2.7 Selection Analysis

**FIGURE 2:**
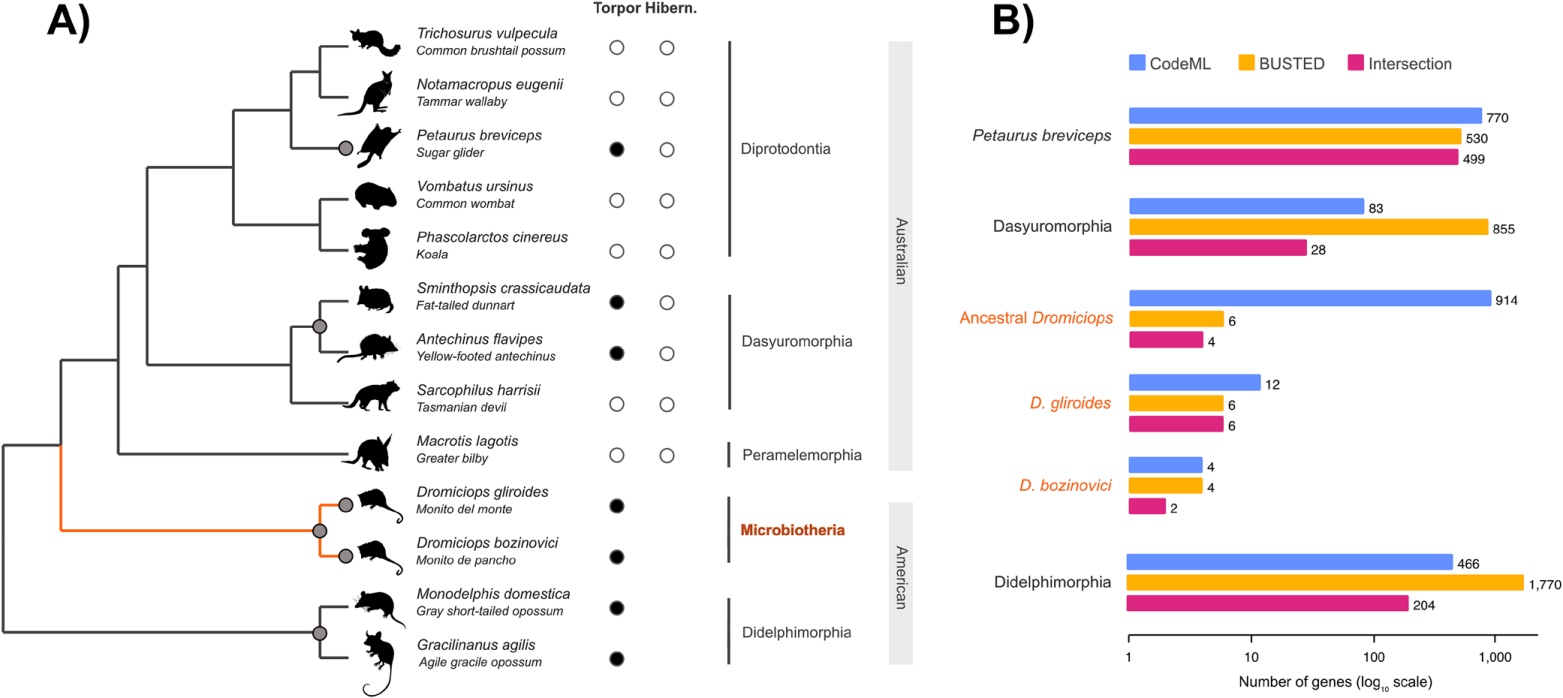
Marsupial topology with torpor and hibernation capacity, and number of genes under positive selection by lineage. (A) Topology of 13 marsupial species representing five orders. Circles on branches and nodes indicate the lineages tested for positive selection in downstream analyses. Filled circles additionally indicate documented capacity for torpor (daily or seasonal) and hibernation; open circles indicate absence of these traits. The torpor capacity was extracted from Geiser and Cooper (2023). (B) Number of genes detected under positive selection in each lineage by CodeML branch‐site model (blue), BUSTED (orange), and their intersection (pink), shown on a log_10_ scale.

To investigate potential genetic mechanisms associated with torpor in *Dromiciops*, we employed a phylogenomic approach including both American and Australian marsupials with varying torpor expression (Figure 2A, Table S2). The classification of exhibiting or not exhibiting torpor was conducted based on the review by Geiser and Cooper (2023). CDS from reference genome sequences were retrieved from NCBI for 11 species across five marsupial orders: Didelphimorphia ( *Gracilinanus agilis* , GCF_016433145.1; *Monodelphis domestica* , GCF_027887165.1), Dasyuromorphia ( *Sminthopsis crassicaudata* , GCF_048593235.1; *Sarcophilus harrisii* , GCF_902635505.1; *Antechinus flavipes* , GCF_016432865.1), Peramelemorphia ( *Macrotis lagotis* , GCF_037893015.1), and Diprotodontia ( *Vombatus ursinus* , GCF_900497805.2; *Petaurus breviceps* , GCF_028583685.1; *Trichosurus vulpecula* , GCF_011100635.1; *Notamacropus eugenii*, GCF_028372415.1; *Phascolarctos cinereus*, GCF_003287225.1). Additionally, coding sequences (CDS) from the *D. gliroides* reference genome (GCF_019393635.1) were obtained for downstream comparative analyses. CDS for all *Dromiciops* genomes were extracted from the GFF of the *D. gliroides* reference genome that was used to perform the assembly (NCBI RefSeq assembly: GCF_019393635.1). The extraction was carried out through AGAT v0.8.0 (https://github.com/NBISweden/AGAT), which was also used to filter by isoform, extracting only the longest. The sex chromosomes and unidentified scaffolds were removed, leaving only the autosomal chromosomes. A single *D. bozinovici* individual was retained for downstream analyses to avoid potential noise introduced by the presence of highly similar sequences. CDS were then used to identify orthogroups through the Orthofinder pipeline v2.5.2 (https://github.com/davidemms/OrthoFinder) (Emms and Kelly 2019). Of the orthogroups obtained, only those that had representation of all the individuals, and that also did not present paralogs, were considered for further analysis. Filtered orthogroups were then aligned by codons using the fas2msa script with default parameters from the GWideCodeML package (Macías et al. 2020). Although this script also removes stop codons if they are present in the sequences, as an additional quality control step, a custom Python script was applied to ensure complete removal of any remaining stop codons prior to downstream analyses. Since branch‐site model analyses require a fixed input topology, a reference phylogeny for the 13 marsupial species was assembled based on established phylogenetic literature, following inter‐ordinal relationships and the placement of Microbiotheria as sister to Euaustralidelphia (Nilsson et al. 2010; Meredith et al. 2008), intra‐ordinal relationships within Dasyuromorphia (Kealy and Beck 2017), and the Diprotodontia framework of Meredith et al. (2009).

A dN/dS test was performed through CodeML (Yang 1997), using the ETE3 algorithm (Huerta‐Cepas et al. 2016). We used the branch‐site model to detect candidate genes for positive selection in the *Dromiciops* genus and in its two species, *D. bozinovici* and *D. gliroides*. Additionally, this analysis was conducted for all lineages associated with torpor‐expressing species (Figure 2A), rotating the setting of each one as foreground and background branches. The branch‐site of CodeML model tests for positive selection acting on a subset of sites in the foreground branch, comparing a null model in which ω is constrained to be ≤ 1 in the foreground branch (model bsA1) against an alternative model that allows *ω* > 1 (model bsA) (Zhang et al. 2005). To reduce the incidence of false positives, *p*‐values obtained from the LRT were corrected for multiple testing using the Benjamini–Hochberg false discovery rate (FDR) procedure. In addition to CodeML, BUSTED (Murrell et al. 2015) from the HyPhy package (Pond et al. 2005) was used to identify candidate genes under positive selection. BUSTED allows the detection of evidence for positive selection at a fraction of sites within a gene while accounting for variation in selective pressures across branches throughout the phylogenetic tree. It uses a likelihood ratio test (LRT) to compare a model that includes positive selection (*ω* > 1 at some sites) with one that does not. As for CodeML, *p*‐values obtained from the LRT were corrected for multiple testing using the Benjamini–Hochberg false discovery rate (FDR) procedure.

### 2.8 Enrichment Analysis

A functional enrichment analysis was then performed using the enrichGO and enrichKEGG functions from the clusterProfiler R package (Wu et al. 2021), to determine whether any pathway or gene ontology biological category was over‐represented among the candidate genes under selection. GO terms assignment was based on the org.Hs.eg.db annotation database for *Homo sapiens* , used as a proxy organism given the absence of a comprehensive functional annotation for *Dromiciops*. Homology between *Dromiciop*s and human genes was established through the human gene symbols assigned to the *D. gliroides* reference genome via homology‐based inference by the NCBI annotation pipeline. The background gene set was defined as the full set of 1:1 orthologous genes used as input for the positive selection analyses, rather than the complete human genome, to avoid inflating enrichment signals with genes not represented in our dataset.

To reduce the redundancy of the enriched Gene Ontology terms, we used the R package *rrvgo* (Sayols 2023), inspired by the approach implemented in REVIGO (Supek et al. 2011). Semantic similarity between GO terms was calculated using the “Rel” method, and terms with a pairwise similarity above 0.7 were grouped into clusters represented by the most semantically significant term.

### 2.9 Protein Family Evolution Analysis

In order to gain further insight into the evolutionary history of *Dromiciops* , gene family expansion and contraction were analyzed. The analysis was conducted using protein‐coding genome sequences from the marsupial species used for the positive selection analyses, including the *D. bozinovici* and *D. gliroides*. To minimize redundancy associated with alternative isoforms, protein datasets from each genome assembly were filtered prior to orthology inference, retaining only the longest isoform per gene using SeqKit (Shen et al. 2016). Orthogroups were identified using the OrthoVenn3 workflow with OrthoFinder, applying an E‐value cutoff of 1 × 10^−10^ and an inflation value of 2.0. The resulting clusters were used to assess gene family expansion. Gene family evolution, including gains and losses, was assessed with CAFE v5 (Mendes et al. 2020), applying the lambda parameter to estimate birth and death rates. Given that the program requires an ultrametric tree, the tree was constructed using single‐copy genes inferred with FastTree2 (Price et al. 2010) and calibrated using the estimated divergence time (64 Mya) between the koala and *Dromiciops*, as provided by TimeTree (Kumar et al. 2022). Heatmaps were generated using row‐scaled orthogroup copy numbers. For each orthogroup, copy numbers were standardized across species using *z*‐score transformation prior to visualization. Consequently, color intensities represent relative enrichment or depletion of gene copies within each orthogroup rather than absolute copy‐number values.

## 3 Results

### 3.1 Demographic History

The demographic history of *Dromiciops* (Figure 1B) reveals contrasting long‐term trajectories between the two species. Based on PSMC analyses (Figure 1B; Figure S1), the split between the two lineages is estimated at approximately 4 million years ago. PSMC results revealed similar patterns of demographic fluctuation for both species, with differences in the magnitude and duration of effective population size changes (Figure 1B). Compared to *D. gliroides* , the two samples of *D. bozinovici* exhibited consistently larger effective population sizes, reaching their highest values between approximately 2 million and 100 thousand years ago. Their demographic trajectories also showed greater variation over time, with more pronounced fluctuations in population size. Within *D. bozinovici*, the Llancalil sample exhibited a marked expansion in effective population size between approximately 500 and 200 thousand years ago. Notably, the Nahuelbuta sample was the only one among all *Dromiciops* individuals analyzed that did not show a noticeable contraction between 200 and 25 thousand years ago.

In contrast to *D. bozinovici*, *D. gliroides* exhibited overall smaller effective population sizes and less pronounced demographic fluctuations throughout the inferred timeline. One exception was the sample from Chonchi, Chiloé Island (*D. gliroides gliroides*), which showed a demographic trajectory that diverged from the general pattern observed in the other *D. gliroides* samples.

As for the recent temporal scale, the contemporary demographic history inferred using the GONE model suggests a prolonged and intense expansion of the effective population size in *D. bozinovici* approximately 200 generations ago, followed by a recent and severe *N*_e_ reduction occurring 6–7 generations ago (Figure S2).

### 3.2 Genomic Diversity

The SNP density analysis revealed differences in genomic diversity between and within *Dromiciops* species (Figure 1C). Figure 1C provides a genome‐wide representation of SNP density variation across chromosome‐length scaffolds, enabling comparison of lineage specific patterns of genomic variation among *Dromiciops* species. In *D. bozinovici* (Figure 1C), higher densities of heterozygous SNVs were observed across the genome compared to *D. gliroides* (Figure 1C). Although all individuals were mapped against the *D. gliroides* reference assembly, higher SNP density was consistently observed in *D. bozinovici* across chromosome‐length scaffolds. Phylogenetic distance between reads and the reference genome could underestimate SNP diversity in resequencing analyses (Galla et al. 2019), which was not the case in our dataset. Within *D. bozinovici*, the sample from Nahuelbuta (Figure 1C) showed slightly higher SNP density than the Llancalil sample (Figure 1C). In *D. gliroides* , lower overall SNP density was observed, with the sample from Chonchi, corresponding to *D. g. mondaca* (Figure 1C), displaying the lowest values among all samples.

### 3.3 Ortholog Identification for Selection Analysis

A total of 21,654 orthogroups were identified for *Dromiciops* and the other marsupial species using Orthofinder, of which 14,446 contained at least one sequence for each taxon evaluated. After filtering, 5022 orthogroups (34.8%) were discarded due to the presence of paralogs. Additionally, 7208 orthogroups were excluded because they lacked sequence representation for one or more taxa. A total of 9424 single‐copy orthogroups (43.5%) were retained for subsequent selection analyses using CodeML and BUSTED.

### 3.4 Candidate Genes for Positive Selection and Enrichment Analysis

**FIGURE 3:**
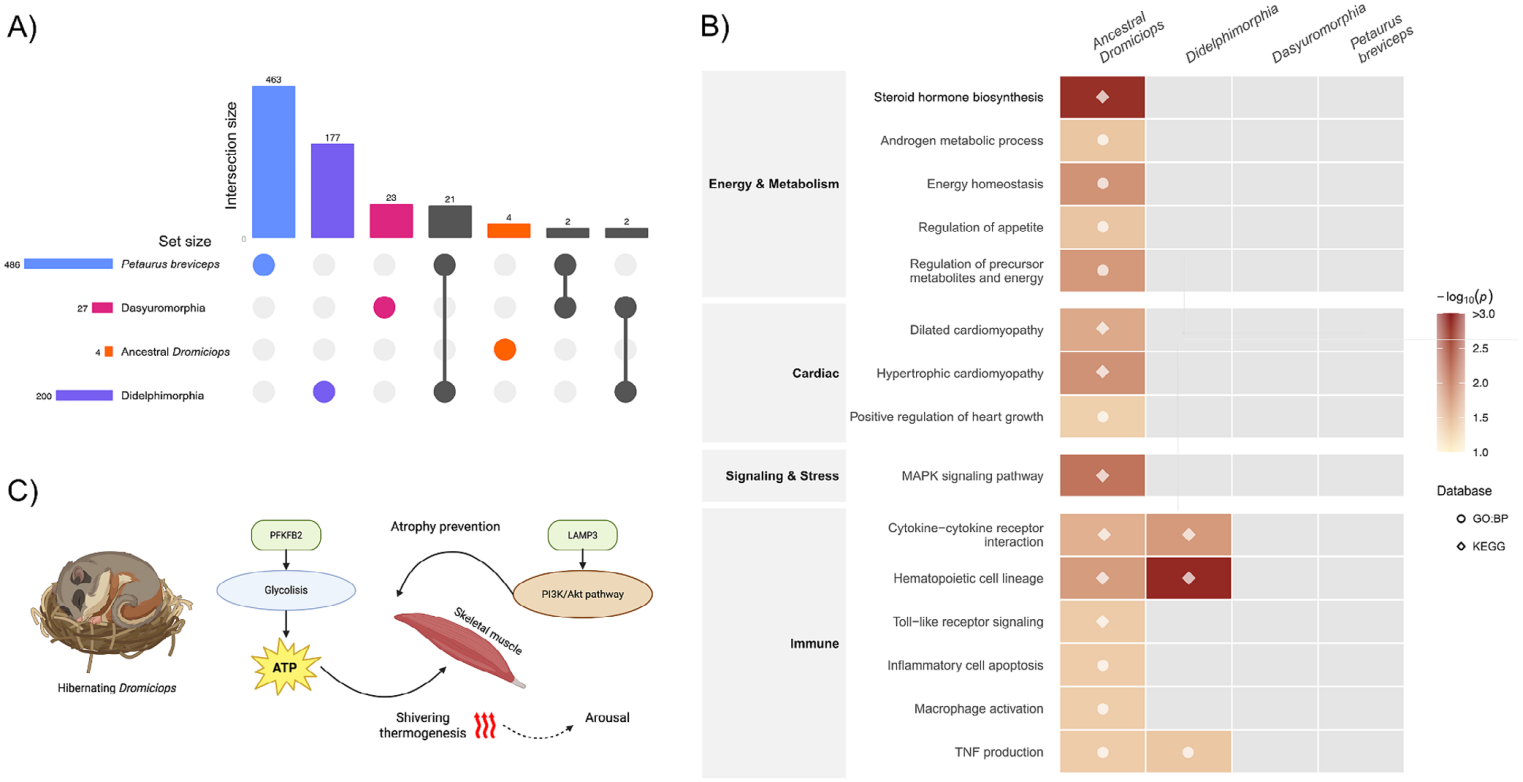
Comparative genomic insights and proposed physiological mechanisms of seasonal torpor exclusivity at the ancestral *Dromiciops* lineage. (A) UpSet plot of positive selection intersections: Visual representation of shared and lineage‐specific candidate genes identified via intersecting CodeML branch‐site and BUSTED frameworks across torpor‐exhibiting marsupial lineages. The ancestral node of *Dromiciops* displays a completely isolated selection profile, sharing zero adaptive gene targets with any other evaluated branch or node. (B) Comparative functional enrichment heatmap: Overrepresented Gene Ontology Biological Process (GO‐BP) terms and KEGG metabolic pathways across the evaluated lineages. The matrix highlights a stark functional divergence, demonstrating that the metabolic and signaling repertoires enriched at the *Dromiciops* ancestral node are predominantly unique and unshared with daily torpor‐bearing marsupials. (C) Schematic model of candidate gene integration in hibernation physiology: Proposed functional network illustrating how positively selected target genes (PFKFB2 and LAMP3) potentially coordinate cellular adaptations in skeletal muscle. Figure created with BioRender.

Following Benjamini–Hochberg FDR correction applied to both CodeML branch‐site and BUSTED results, cross‐referencing the remaining candidates yielded an intersection of four genes (Figure 3A) at the ancestral *Dromiciops* node (*LAMP3*, *OTX1*, *PFKFB2*, *RAPSN*), six genes at the *D. gliroides* branch (*LAMP3*, *OTX1*, *PFKFB2*, *RAPSN*, *LSS*, *RASL11B*), and two genes at the *D. bozinovici* branch (*FLNB*, *HSPA2*); the number of candidates identified by each method across all remaining lineages is summarized in Table S3. Among the *Dromiciops* species intersection genes, *FLNB* was the only one also identified under positive selection in other marsupial orders, while the remaining candidates were specific to the Microbiotheria lineage. Given the limited statistical power of enrichment analyses over such small gene sets, the broader CodeML branch‐site candidate list was used as input for subsequent functional analyses.

Using the CodeML branch‐site candidates after FDR correction as input, the *rrvgo* package in R identified 50 representative biological processes for the ancestral node of *Dromiciops* (Table S4). These include energy homeostasis (GO:0097009), regulation of generation of precursor metabolites and energy (GO:0043467), regulation of appetite (GO:0032098), androgen metabolic process (GO:0008209), positive regulation of heart growth (GO:0060421), positive regulation of MAPK cascade (GO:0043410), forebrain development (GO:0030900), and macrophage activation (GO:0042116), along with terms related to nephron morphogenesis (GO:0072028), osteoblast differentiation (GO:0001649), and tumor necrosis factor production (GO:0032640). Additionally, 11 KEGG‐enriched pathways were identified for the node, including steroid hormone biosynthesis (hsa00140), MAPK signaling pathway (hsa04010), cytokine‐cytokine receptor interaction (hsa04060), pathways associated with hypertrophic and dilated cardiomyopathy (hsa05410 and hsa05414, respectively), Toll‐like receptor signaling (hsa04620), and hematopoietic cell lineage (hsa04640) (Table S5).

The rrvgo package in R identified 12 representative biological processes for *D. bozinovici* (Table S6), based on the set of genes identified using CodeML branch‐site. These include response to cold (GO:0009409), protein refolding (GO:0042026), modulation of excitatory postsynaptic potential (GO:0098815), regulation of postsynaptic membrane potential (GO:0060078), RNA splicing (GO:0008380), mRNA splice site recognition (GO:0006376), cellular response to type II interferon (GO:0071346), and germ cell development (GO:0007281) (Figure 3). Additionally, the KEGG pathway antigen processing and presentation (hsa04612) was found to be enriched in this species.

Similarly, for *D. gliroides* , 7 biological processes were identified as representative by rrvgo (Table S7), based on the set of genes identified using CodeML branch‐site. These processes include regulation of protein stability (GO:0031647), plasma membrane repair (GO:0001778), glucose catabolic process (GO:0006007), brain morphogenesis (GO:0048854), regulation of cation channel activity (GO:2001257), secondary alcohol biosynthetic process (GO:1902653), and cholinergic synaptic transmission (GO:0007271). Additionally, one KEGG pathway was identified for *D. gliroides* , fructose and mannose metabolism (hsa00051). Enriched biological processes for the remaining evaluated nodes can be found in Table S8.

### 3.5 Gene Family Expansion/Contraction

**FIGURE 4:**
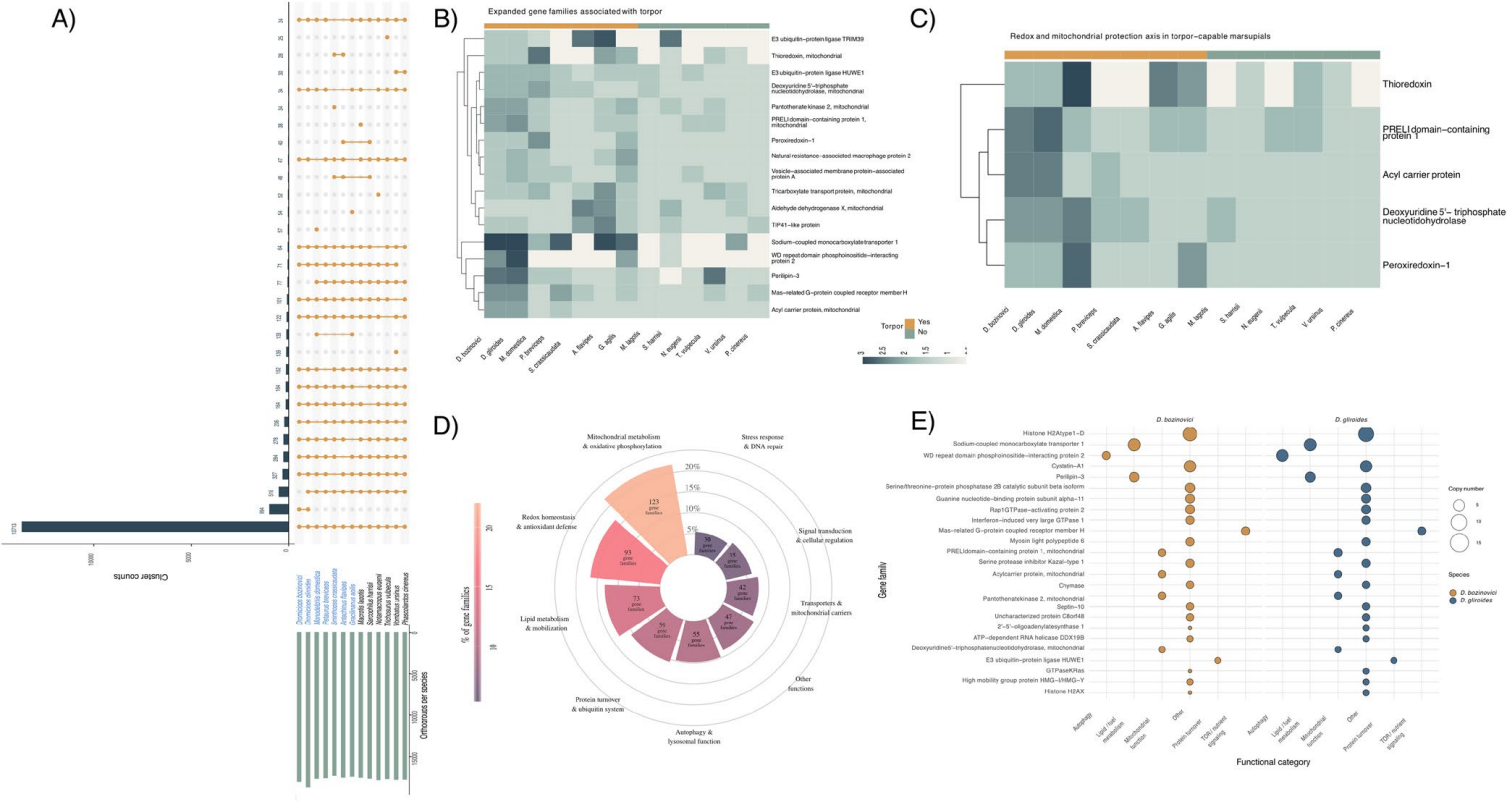
Orthogroup overlap and recurrent gene‐family expansions associated with torpor in marsupials. (A) Orthogroups across marsupial genomes. Torpor‐associated species include *Dromiciops gliroides* , *D. bozinovici*, *Antechinus flavipes* , *Gracilinanus agilis* , *Sminthopsis crassicaudata* , and *Petaurus breviceps* (names in blue color) and in black color are the names of not torpor‐associated species. (B) Recurrently expanded multicopy orthogroups associated with torpor‐capable marsupials. For heatmap visualization, color scale represents row‐standardized (*z*‐score) orthogroup copy numbers. (C) Expanded orthogroups associated with mitochondrial metabolism, redox regulation, oxidative stress buffering, and mitochondrial maintenance pathways. Candidate families include thioredoxin mitochondrial (TXN2; cluster808), peroxiredoxin‐1 (PRDX1; cluster926) among others. The percentages shown in D represent the proportion of expanded gene families assigned to each functional category relative to the total number of expanded gene families identified. (D) Functional categorization of recurrently expanded orthogroups in *Dromiciops*. Categories were assigned based on curated functional annotations and grouped into major biological processes associated with mitochondrial metabolism, oxidative phosphorylation, lipid metabolism, stress response, intracellular signaling, autophagy, and cellular maintenance. (E) Functionally annotated expanded gene families in *Dromiciops* species. Shared and lineage‐biased expansions between *D. gliroides* and *D. bozinovici* are shown.

Orthology inference across the marsupial genomes identified 20,547 orthogroups from a total of 272,753 predicted proteins, including 8900 single‐copy orthogroups and 5071 singleton proteins, identified for only one species (1.86% of all proteins analyzed) (Table S9). The number of orthogroups per species ranged from 17,262 to 18,690, whereas singleton counts varied substantially among assemblies. Comparative overlap analyses revealed 13,713 shared orthogroups across the selected species dataset (Figure 4A).

Comparative analyses across marsupial genomes identified recurrent differences in orthogroup copy number between species reported to exhibit torpor and species without documented torpor behavior. UpSet analyses revealed both shared and lineage‐specific orthogroups among torpor‐associated marsupials (Figure 4A). Orthogroups were recurrently expanded across multiple torpor species, whereas non‐torpor species generally showed lower copy numbers for these same families. Expanded orthogroups in torpor‐associated species involved genes linked to mitochondrial metabolism, oxidative phosphorylation, redox regulation, lipid metabolism, and intracellular signaling (Figure 4B; Figure S3). Among them, highlight a few such as thioredoxin mitochondrial (TXN2; cluster808), peroxiredoxin‐1 (PRDX1; cluster926), mitochondrial acyl carrier proteins involved in complex I assembly (cluster957), and mitochondrial dUTPase‐related families (cluster771) (Figure 4C). In contrast, several highly expanded olfactory receptor families were also detected but were analyzed separately due to their large copy‐number variability across marsupials (Figure S4).

Functional categorization of expanded orthogroups of the *Dromiciops* genus showed a predominance of genes associated with electron transport chain activity, mitochondrial function, oxidative phosphorylation, and respiratory metabolism (Figure 4D). Additional candidates were linked to lipid metabolism, autophagy, mitochondrial dynamics, and stress‐response pathways.

When the two *Dromicips* species were compared the analyses revealed expanded shared multicopy orthogroups associated with mitochondrial function and redox homeostasis. Several of these candidates were either absent or present at lower copy numbers in non‐torpor marsupials, suggesting a potential association between copy‐number expansion and physiological mechanisms related to metabolic suppression and oxidative stress tolerance (Figure 4E). Despite this shared pattern, both species also exhibited lineage‐biased expansions. *D. gliroides* showed relatively higher copy numbers in families associated with energy metabolism and nutrient‐related pathways, whereas *D. bozinovici* showed comparatively stronger expansion of stress‐response and mitochondrial protection‐related families.

## 4 Discussion

Our results suggest that the evolutionary history of *Dromiciops* has been shaped by adaptive processes operating at two distinct levels. At the macroevolutionary level, positive selection and gene family expansions at the ancestral node converge on mitochondrial function, oxidative stress regulation, and metabolic flexibility—processes broadly consistent with the physiological demands of torpor and hibernation—supporting the hypothesis that this capacity has been a long‐standing selective target in the lineage. At the species level, the two extant species show no overlap in their positively selected genes and appear to have followed divergent molecular trajectories: *D. gliroides* presents signatures associated with carbohydrate metabolism and lipid remodeling, potentially linked to its greater reliance on seasonal fleshy‐fruited resources in southern temperate forests, while *D. bozinovici* shows evidence of selection on thermal stress tolerance, consistent with its occupation of environments characterized by wider thermal amplitude. Together, these patterns suggest that, while torpor physiology may represent a shared and ancestrally selected trait in *Dromiciops*, the molecular mechanisms underlying adaptation in each lineage are largely lineage‐specific—a finding consistent with emerging evidence that torpor has evolved through distinct genomic solutions across mammalian lineages.

Historical climate events may have influenced geographical distribution and fragmentation of *Dromiciops* populations, likely exerting strong selection pressures and potentially driving lineage divergence approximately 4 Mya as shown in the PSMC (Figure 1B) and similar dates estimated around 3 Mya for the species split using RADseq data (Quintero‐Galvis et al. 2022). Differences in genomic diversity among populations appear consistent with their inferred demographic trajectories (Figure 1C; Figure S1): populations with larger historical *N*_e_ maintained higher heterozygosity in northern regions, while those with signs of recent bottlenecks exhibited reduced variation in southern regions. However, demographic responses were not uniform across lineages, suggesting that glacial cycles affected populations differently depending on local refugia, geographic isolation, and lineage‐specific histories, particularly within long‐term forest refugia of the Chilean Coastal Range (Premoli et al. 2019). The larger effective population size observed in *D. bozinovici* seems to occur as a result of its distribution in northern regions, which were located outside the limits of ice sheet expansion during the Pleistocene glaciations (Harrison 2004; Heusser 2003). In contrast, the southernmost *D. gliroides* exhibited low effective population sizes followed by a post‐LGM increase (Figure 1B; Figure S2), a pattern that combined with its reduced genomic diversity is consistent with a recent colonization event and a potential founder effect from the mainland (Himes et al. 2008).

These contrasting histories and genomic diversity patterns between the two species, shaped by different levels of isolation, population size, and habitat stability, provide a relevant context for interpreting divergent adaptive responses within and between *Dromiciops* lineages. Superimposed on these long‐term dynamics, GONE reconstructions revealed a marked recent population decline in *D. bozinovici* (Figure S2; Table S1), likely associated with the large‐scale deforestation of temperate rainforests and their replacement by commercial plantations (Echeverría et al. 2006; Fontúrbel et al. 2022); the loss of key structural elements such as suitable nesting substrates may have intensified this trend (Fontúrbel et al. 2022), further shaping the demographic context in which adaptive processes have unfolded.

Beyond lineage differentiation, the selective pressures derived from ancestral climatic conditions may also have favored the evolution of adaptive strategies enabling *Dromiciops* to persist under extreme environmental variation. This mechanism helps organisms cope with unpredictable and adverse conditions, such as low temperatures and food scarcity (Mohr et al. 2020; Bozinovic et al. 2004). We identified candidate genes under positive selection and gene family expansions that could be associated with stress response, torpor, and hibernation, highlighting the adaptive relevance of this phenotype in *Dromiciops*' evolutionary history.

Four genes under positive selection at the ancestral *Dromiciops* node (*LAMP3*, *OTX1*, *PFKFB2*, and *RAPSN*) were recovered consistently across CodeML branch‐site and BUSTED analyses and were absent from all other torpor‐bearing marsupial lineages examined (Figure 3A). Similarly, the majority of enriched pathways identified for the ancestral node were not observed in the other marsupials evaluated (Figure 3B). This is noteworthy given that *Dromiciops* is the only marsupial in our dataset that exhibits seasonal torpor or hibernation, as opposed to the daily torpor characteristic of the other lineages. The exclusivity of this pattern could reflect at least three non‐mutually exclusive explanations: characteristics specific to the Microbiotheria lineage unrelated to torpor; general physiological processes not specific to hibernation; or functions specifically linked to seasonal, rather than daily, torpor. Although none of these hypotheses can be resolved with the present data, some of the identified genes and metabolic processes are consistent with known aspects of torpor physiology.

Two positively selected genes at this node (*PFKFB2* and *LAMP3*) present particularly relevant functional connections. *PFKFB2* encodes a key regulator of glycolytic flux, whose product is the primary activator of the rate‐limiting enzyme of glycolysis, PFK‐1 (Ros and Schulze 2013). Beyond its role in routine ATP production, glycolysis becomes the predominant energy source under hypoxic conditions—such as those experienced during hibernation—where reduced oxygen availability limits oxidative phosphorylation and forces a greater dependence on anaerobic ATP generation (Kadamani et al. 2024; Ros and Schulze 2013). This is particularly relevant given the energetic demands of shivering thermogenesis—the primary source of heat production in hibernating marsupials given their lack of functional brown adipose tissue (Hayward and Lisson 1992; Polymeropoulos et al. 2012)—and its direct role in rewarming during arousal from torpor in this group (Hadj‐Moussa et al. 2016; Opazo et al. 1999; Geiser and Cooper 2023). Additionally, *PFKFB2* exerts cardioprotective effects under hypoxia in rodents (Gao et al. 2021), a condition also arising during hibernation, and is overexpressed in skeletal muscle during acute cold exposure (Dey et al. 2025), suggesting its involvement in a broader metabolic response to thermal stress of which hibernation represents an extreme case.

*LAMP3*, in turn, participates in autophagy (Nagelkerke et al. 2014) and in lipogenesis through activation of the PI3K/Akt pathway (Liao et al. 2018), preventing muscle atrophy in rodents (Stitt et al. 2004), which is a relevant adaptation to counteract disuse‐induced muscle loss during hibernation. It also modulates the transition between carbohydrate and lipid utilization throughout torpor, ensuring efficient energy management under prolonged metabolic suppression (Tessier et al. 2015). Together, *PFKFB2* and *LAMP3* present functional connections consistent with the physiological demands of prolonged torpor in *Dromiciops* (Figure 3C), although their direct involvement will require experimental validation.

Several enriched biological pathways at the ancestral *Dromiciops* node further support a potential link to torpor and hibernation. Two stand out as particularly relevant: steroid hormone biosynthesis and the MAPK signaling pathway, also supported by the enrichment of the biological process positive regulation of MAPK cascade. Although both are broadly conserved pathways with diverse physiological functions, each has been previously implicated in hibernation physiology in mammals (Wijenayake et al. 2018; Eddy and Storey 2007; Boonstra et al. 2011).

The MAPK pathway coordinates cellular responses including proliferation, differentiation, stress response, and apoptosis (Eddy and Storey 2007; Zhu et al. 2005). Crucially, it was also identified as enriched in a torpor‐related transcriptomic analysis of *D. gliroides* itself (Wijenayake et al. 2018), strengthening the case for its functional relevance in this lineage. Its three principal cascades—ERK, JNK, and p38—are activated differentially across organs and species: in bats, p38 MAPK activation in skeletal muscle may help prevent muscle atrophy during torpor (Eddy and Storey 2007), while in Arctic ground squirrels, all three cascades may contribute to energy balance in brain and heart during this state (Zhu et al. 2005).

The enrichment of the steroid hormone biosynthesis is similarly suggestive: adrenal androgen production prior to hibernation has been associated with muscle mass accumulation in Arctic ground squirrels (Boonstra et al. 2011), and glucocorticoids have been implicated in energy mobilization and arousal physiology in hibernating bats (Willis and Wilcox 2014), suggesting that steroid hormone regulation may be a conserved component of mammalian hibernation physiology.

Although the number of biological processes shared under positive selection between *Dromiciops* and other torpor‐bearing marsupials was low, gene family analyses identified recurrent expansion of multicopy orthogroups in torpor‐associated species—including gene families linked to mitochondrial maintenance, redox regulation, and oxidative stress buffering—processes previously implicated in mammalian hibernation physiology (Carey et al. 2003; Giroud et al. 2021). Independently, positive selection analyses recovered candidate genes and enrichment profiles related to energy homeostasis, metabolic regulation, and cellular stress tolerance at the ancestral *Dromiciops* node. Although overlap between the two approaches was limited at the gene level, both converged on mitochondrial function and redox homeostasis, suggesting coordinated genomic signatures linked to metabolic suppression and torpor physiology, and pointing to the recurrent expansion of these families as a potentially conserved component of metabolic depression across torpor‐capable lineages.

The limited overlap in positively selected genes across torpor‐bearing marsupials, alongside the broader patterns of gene family expansion, suggests that convergent adaptation to torpor operates through at least two distinct evolutionary mechanisms: lineage‐specific positive selection shaping distinct molecular solutions, and shared expansions of gene families reflecting sustained selective pressure on metabolic flexibility. This is consistent with emerging evidence that torpor has evolved through lineage‐specific genomic solutions across distantly related mammals (Mohr et al. 2020; M. T. Andrews 2019)—even where shared gene expression programs exist across divergent lineages (Weir et al. 2024). Whether the positively selected genes identified here in *Dromiciops* are functionally analogous to those related to torpor regulation in other lineages remains an open and compelling question for future functional studies. Beyond these ancestral signatures, the two extant *Dromiciops* species show no overlap in positively selected genes under either analytical framework, suggesting divergent molecular trajectories since their separation.

At the *D. gliroides* branch, the same four positive selection candidate genes identified at the ancestral node (*LAMP3*, *OTX1*, *PFKFB2*, and *RAPSN*) were recovered, alongside two additional candidates: *LSS* and *RASL11B*. *LSS* encodes a key enzyme in cholesterol biosynthesis linked to membrane fluidity maintenance at low body temperatures (Ruf and Geiser 2015). *RASL11B*, a member of the RAS GTPase superfamily, has been implicated in the suppression of apoptotic signaling under cellular stress, a function critical for preserving tissue integrity during prolonged metabolic depression (Storey and Storey 2004). Functional enrichment analyses were consistent with these gene‐level signals, revealing enrichment in lipid and sterol biosynthetic processes (GO:1902653, GO:0008299) as well as the KEGG pathway for fructose and mannose metabolism (hsa00051)—a result particularly notable given the identification of *PFKFB2*, a central regulator of carbohydrate metabolism, at both the ancestral and *D. gliroides* nodes.

Notably, independent support for the relevance of carbohydrate processing in *D. gliroides* comes from a population genomic study based on RAD‐seq data, which identified ALG8—a glucosyltransferase involved in glycoprotein glycosylation—as the only locus consistently detected under selection by all five analytical methods applied to this species (Quintero‐Galvis et al. 2024). Although ALG8 and PFKFB2 participate in distinct metabolic pathways, the convergence of two independent genomic approaches on sugar‐related processes suggests that carbohydrate metabolism represents an important axis of molecular differentiation in *D. gliroides* .

This interpretation gains additional ecological context from recent species distribution models integrating trophic interactions in *Dromiciops* (Pizarro et al. under review), which showed that the main fleshy‐fruited plant species associated with *Dromiciops* foraging (Amico et al. 2009) are predominantly distributed across the southern temperate rainforests occupied by *D. gliroides* , while being comparatively less represented across the range of *D. bozinovici*. Given that *Dromiciops* relies heavily on seasonal fruit consumption to sustain energetic demands associated with torpor and pre‐hibernation fat accumulation, spatial differences in carbohydrate‐rich food availability may impose distinct energetic and ecological pressures across species distributions.

At the *D. bozinovici* branch, the intersection of methods identified two positive selection candidate genes: *FLNB* and *HSPA2*. *HSPA2*, a member of the *HSP70* molecular chaperone family, functions in protein quality control during thermal perturbation, and has been implicated in the cellular response to temperature stress across multiple taxa (Wang et al. 2015; Vydra et al. 2009; Mohanarao et al. 2014; Xu et al. 2017)—though the nature of this response varies by tissue and stress type. The three positively selected sites identified by CodeML (positions 154, 164, and 171) fall within the ATPase domain of the protein, which drives the conformational cycle underlying chaperone activity. This pattern contrasts with the primary selection signal in avian HSPA2, concentrated in the peptide‐binding domain and linked to adaptation to spermatogenesis at elevated internal body temperatures (Padhi et al. 2016), suggesting that selection in *D. bozinovici* targets the regulation or efficiency of the chaperone cycle across a wider thermal range rather than substrate specificity. The enrichment profile was consistent with this interpretation, showing enrichment in response to cold (GO:0009409) and protein refolding (GO:0042026). Notably, *D. bozinovici* occupies the northernmost range of the genus, including populations at higher elevations in the Andes and Coastal Cordillera (Quintero‐Galvis et al. 2024), where thermal amplitude is considerably greater than in the more climatically buffered range of *D. gliroides*—a distributional pattern that may help explain why selection in this lineage appears to target broad thermal stress tolerance rather than cold adaptation alone. It has been suggested that *Dromiciops* may also undergo estivation in response to high ambient temperatures (Fonturbel et al. 2022), which, if confirmed, would further support this interpretation.

## 5 Conclusion

The identification at the ancestral node of a restricted number of genes under positive selection (with convergent support from both analyses) and expanded gene families potentially linked to torpor and hibernation exclusively within the order Microbiotheria highlights the significance of these mechanisms in shaping its evolutionary strategy, allowing *Dromiciops* to thrive in extreme and fluctuating environmental conditions. A significant number of studies investigating the genetic basis of hibernation focus on evaluating differentially expressed genes during this process. However, relatively few have examined the influence of positive selection over the evolution of torpor and hibernation. Hibernation, as a response to harsh environmental conditions, depends on the coordinated regulation of various metabolic and physiological pathways. Our results reveal key pathways enriched through positive selection and gene expansion analyses, including several metabolic pathways and biological processes related to muscle contraction and maintenance, which appear to play a crucial role in sustaining homeostasis and promoting thermogenesis during this process. Furthermore, we identified several species‐specific candidate genes under positive selection, suggesting that each *Dromiciops* species may have undergone distinct evolutionary pressures leading to unique adaptations. However, it is important to note that recent adaptive differences may not have been fully captured by our analyses. Therefore, additional approaches, such as population level studies, are recommended to assess potential local adaptations of each species. The identification of several candidate genes under positive selection unique to each *Dromiciops* species, along with differences in demographic history and genetic diversity, further supports their recent taxonomic delimitation. Altogether, these results highlight the role of natural selection in driving the evolutionary and ecological divergence of *Dromiciops* species and provide valuable insights into the genetic mechanisms underlying hibernation and other adaptive traits in this genus.

## Author Contributions

**Elisa Gonzalez‐Ugalde:** data curation (equal), formal analysis (equal), investigation (equal), methodology (equal), writing – original draft (equal), writing – review and editing (equal). **Paula M. Avendaño:** data curation (equal), formal analysis (equal), investigation (equal), methodology (equal), writing – original draft (equal), writing – review and editing (equal). **Julián F. Quintero‐Galvis:** formal analysis (equal), investigation (equal), methodology (equal), writing – review and editing (equal). **Eduardo J. Pizarro:** data curation (equal), formal analysis (equal), investigation (equal), methodology (equal), writing – review and editing (equal). **Francisco A. Cubillos:** funding acquisition (equal), investigation (equal), writing – review and editing (equal). **Roberto F. Nespolo:** funding acquisition (equal), investigation (equal), writing – review and editing (equal). **Fabiola León:** investigation (equal), methodology (equal), supervision (equal), validation (equal), visualization (equal), writing – original draft (equal), writing – review and editing (equal). **Juliana A. Vianna:** conceptualization (equal), data curation (equal), formal analysis (equal), funding acquisition (equal), investigation (equal), methodology (equal), project administration (equal), supervision (equal), validation (equal), visualization (equal), writing – original draft (equal), writing – review and editing (equal).

## Funding

This work was supported by Agencia Nacional de Investigación y Desarrollo de Chile—Programa Iniciativa Milenio (NCN2024_040—LILI, ICN2021_044—CGR, ICN2021_002—BASE).

## Conflicts of Interest

The authors declare no conflicts of interest.

## Supporting information


**Figure S1:** Bootstrap PSMC analysis for five *Dromiciops* genomes from different localities. Each panel represents 100 bootstrap replicates of historical effective population size (*N*_e_) over time, generated using the PSMC model. The solid red line corresponds to the original PSMC estimate, while light red lines show the bootstrap replicates, indicating the confidence interval of *N*_e_ estimates. (a) *D. bozinovici* from Nahuelbuta, (b) *D. bozinovici* from Llancalil, (c) *D. gliroides gliroides* from San Martín, (d) *D. gliroides gliroides* from Llancahue, and (e) *D. gliroides mondaca* from Chonchi (Chiloé Island). All reconstructions assumed the same generation time and mutation rate per generation.
**Figure S2:** GONE; recent demographic history of *D. bozinovici* inferred by high‐resolution analysis of linkage disequilibrium.
**Figure S3:** Expanded multicopy orthogroups identified across marsupial species reported to exhibit torpor.
**Figure S4:** Olfactory receptor family expansions across marsupial genomes. Copy‐number variation of highly expanded olfactory receptor‐associated orthogroups across marsupial genomes. These families were analyzed separately due to their extreme copy number variability and large contribution to overall orthogroup expansion patterns.

**Table S1:** Detailed information on the five Dromiciops individuals used in this study, including sampling localities, taxonomic identification, and tissue type. These samples were used for genome sequencing and subsequent demographic and adaptive genomic analyses.
**Table S2:** Taxonomic classification, geographical distribution, and sequence accessions for the 11 marsupial species retrived from NCBI. Columns display the corresponding Order, Family, Scientific Name, and Geographical Distribution for each lineage. NCBI RefSeq accession numbers are provided for the reference genomes utilized in the coding sequence (CDS) extraction. The final column categorizes each species based on the documented presence or absence of torpor expression and was extracted from the review by Geiser and Cooper (2023).
**Table S3:** Candidate genes under positive selection identified across the evaluated marsupial lineages. Asterisk (*) indicate candidate genes whose adaptive signals were non‐exclusive to the order Microbiotheria.
**Table S4:** Representative Gene Ontology Biological Process (GO‐BP) terms summarized by rrvgo from candidate genes under selection in the Dromiciops ancestral node. The underlying gene set was identified utilizing codeML branch‐site.
**Table S5:** Enriched KEGG pathways from candidate genes under selection in the Dromiciops ancestral node. The underlying gene set was identified utilizing codeML branch‐site.
**Table S6:** Representative Gene Ontology Biological Process (GO‐BP) terms summarized by rrvgo from candidate genes under selection in *Dromiciops bozinovici*. The underlying gene set was identified utilizing codeML branch‐site.
**Table S7:** Representative Gene Ontology Biological Process (GO‐BP) terms summarized by rrvgo from candidate genes under selection in the *Dromiciops gliroides* . The underlying gene set was identified utilizing codeML branch‐site.
**Table S8:** Representative Gene Ontology Biological Process (GO‐BP) terms summarized by rrvgo from candidate genes under selection in the rest marsupials that exhibit torpor. The underlying gene set was identified utilizing codeML branch‐site.
**Table S9:** Orthology inference statistics across marsupial genomes. Summary statistics of orthology inference analyses across the 13 marsupial genomes. The table includes the total number of predicted proteins, proteins assigned to orthogroups, singleton proteins, total orthogroups per species, and global orthology statistics generated from OrthoVenn3 analyses.

## Data Availability

*Dromiciops* raw fastq reads have been deposited in the GenBank database (BioSample accession numbers SAMN53758112 to SAMN53758116). The code needed to replicate the results is available at https://github.com/elisagou/Dromiciops‐codes.
