# Supplementary material for: Frozen and Scorched: Genomic Signatures of Adaptive Divergence and Hibernation in the Living Fossil Dromiciops
Source: Ecol Evol. 2026 Aug 2;16(8):e74066. doi: 10.1002/ece3.74066 (PMC13429303; doi:10.1002/ece3.74066)
Supplement: Supplementary file 1 — Figure S1: Bootstrap PSMC analysis for five Dromiciops genomes from different localities. Each panel represents 100 bootstrap replicates of historical effective population size (N e) over time, generated using the PSMC model. The solid red line corresponds to the original PSMC estimate, while light red lines show the bootstrap replicates, indicating the confidence interval of N e estimates. (a) D. bozinovici from Nahuelbuta, (b) D. bozinovici from Llancalil, (c) D. gliroides gliroides from San Martín, (d) D. gliroides gliroides from Llancahue, and (e) D. gliroides mondaca from Chonchi (Chiloé Island). All reconstructions assumed the same generation time and mutation rate per generation. Figure S2: GONE; recent demographic history of D. bozinovici inferred by high‐resolution analysis of linkage disequilibrium. Figure S3: Expanded multicopy orthogroups identified across marsupial species reported to exhibit torpor. Figure S4: Olfactory receptor family expansions across marsupial genomes. Copy‐number variation of highly expanded olfactory receptor‐associated orthogroups across marsupial genomes. These families were analyzed separately due to their extreme copy number variability and large contribution to overall orthogroup expansion patterns. [file ECE3-16-e74066-s002.docx]

**Supplementary Material. Figures.**

**Frozen and scorched: Genomic signatures of adaptive divergence and hibernation in the living fossil *Dromiciops***

E. González-Ugalde^a,b,c,d*^, P. M. Avendaño^a,b,c,d*^, J.F. Quintero-Galvis^c,e^, E. J. Pizarro, F. ^a,b,c,d^, A. Cubillos^c,f,g^, R. F. Nespolo^c,h,l^, F. León^a,b,c,d^**, J. A Vianna^b,c,d,j^**

**Figure S1:** Bootstrap PSMC analysis for five *Dromiciops* genomes from different localities. Each panel represents 100 bootstrap replicates of historical effective population size (Ne) over time, generated using the PSMC model. The solid red line corresponds to the original PSMC estimate, while light red lines show the bootstrap replicates, indicating the confidence interval of Ne estimates. (a) *D. bozinovici* from Nahuelbuta, (b) *D. bozinovici* from Llancalil, (c) *D. gliroides gliroides* from San Martín, (d) *D. gliroides gliroides* from Llancahue, and (e) *D. gliroides mondaca* from Chonchi (Chiloé Island). All reconstructions assumed the same generation time and mutation rate per generation.


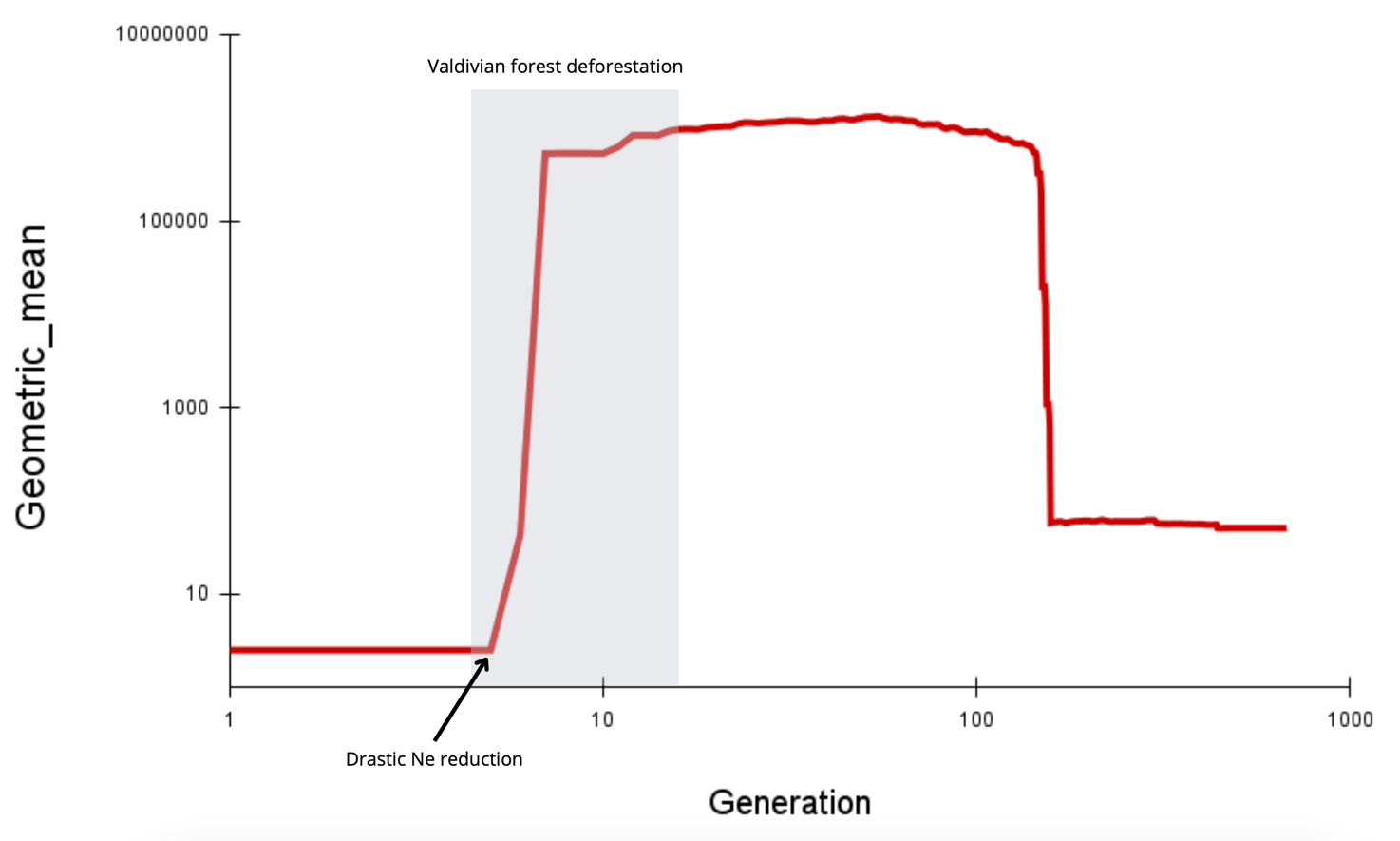


**Figure S2:** GONE; recent demographic history of *D. bozinovici* inferred by high-resolution analysis of linkage disequilibrium.





**Figure S3:** Expanded multicopy orthogroups identified across marsupial species reported to exhibit torpor.


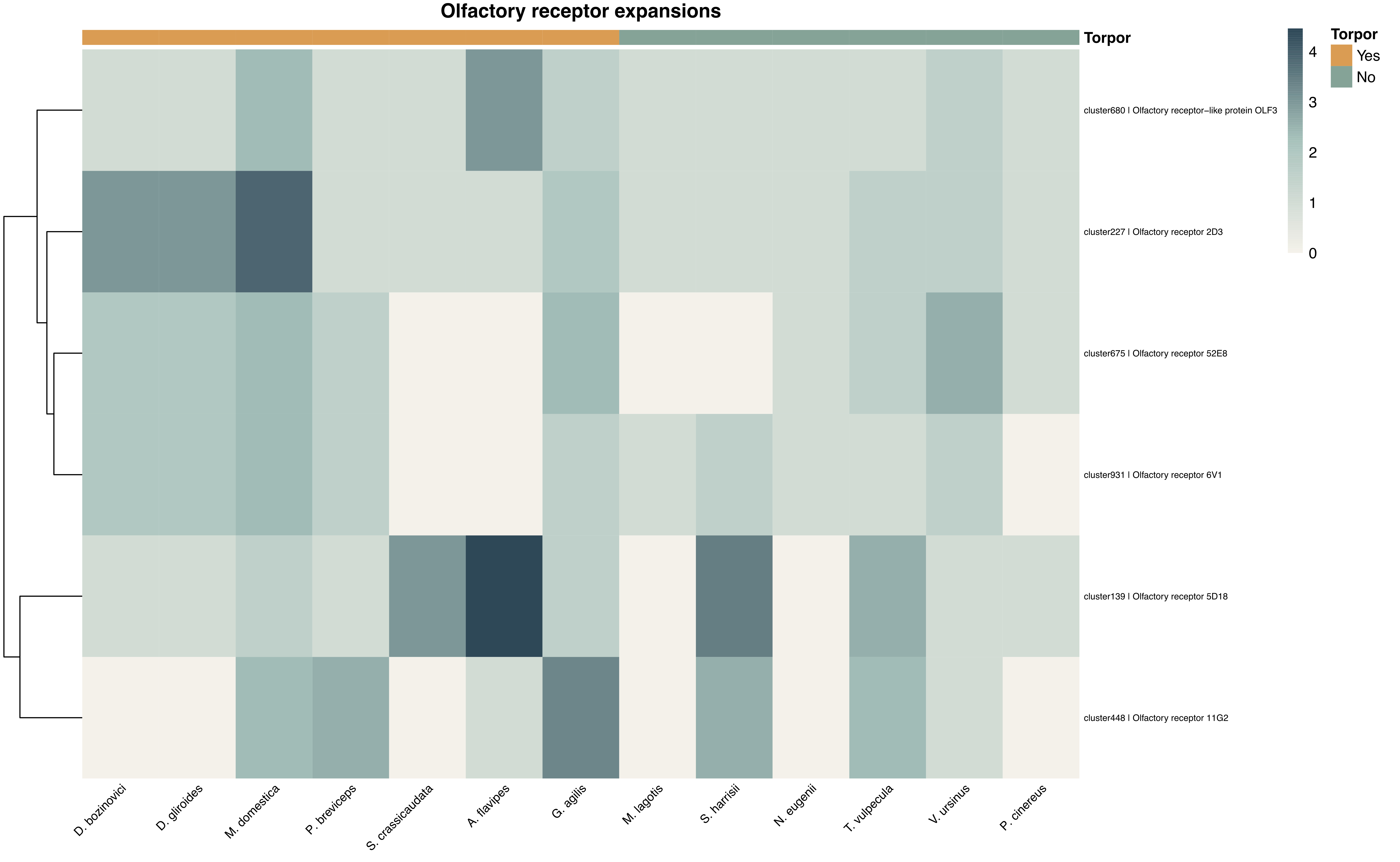


**Figure S4:** Olfactory receptor family expansions across marsupial genomes. Copy-number variation of highly expanded olfactory receptor-associated orthogroups across marsupial genomes. These families were analyzed separately due to their extreme copy number variability and large contribution to overall orthogroup expansion patterns.
